# Statins combined with niacin reduce the risk of peripheral neuropathy

**DOI:** 10.3892/ijfn.2020.3

**Published:** 2020-06-09

**Authors:** STEVEN LEHRER, PETER H. RHEINSTEIN

**Affiliations:** 1 Department of Radiation Oncology, Icahn School of Medicine at Mount Sinai, New York, NY 10029; 2 Severn Health Solutions, Severna Park, MD 21146, USA

**Keywords:** statin, niacin, neuropathy, MedWatch

## Abstract

Statins are a class of lipid-lowering medications that reduce illness and mortality in those who are at a high risk of developing cardiovascular disease. They are the most common cholesterol-lowering drugs. A case control study published in 2002 indicated that statins may increase the risk of peripheral neuropathy. Statin users were 14-fold more likely to develop peripheral neuropathy than non-users, although the overall risk of developing neuropathy was minimal. However, a number of other studies have produced conflicting results regarding neuropathy and statins. Statins are frequently combined with niacin (vitamin B3). Due to its beneficial effects on lipid profiles, niacin has been prescribed for the prevention of heart disease for >40 years. Among the B vitamins, niacin has long been recognized as a key mediator of neuronal development and survival, and may be of value for the treatment of neuropathy. The present study aimed to assess whether the combination of niacin and statin may reduce the risk of peripheral neuropathy attributed to statins. For this purpose, data from MedWatch, the Food and Drug Administration (FDA) Safety Information and Adverse Event Reporting Program were analyzed. The online tool OpenVigil 2.1 was used to query the databases. The results revealed that the majority of statins alone were related to neuropathy. Pitavastatin was the only exception. The association with neuropathy was most pronounced in the lipophilic statins: Atorvastatin and fluvastatin. The association was weaker for other lipophilic statins, such as lovastatin and simvastatin. Two hydrophilic statins, rosuvastatin and pravastatin, exhibited a similarly weaker association with neuropathy, while no reports of any association of pitavastatin with neuropathy were found. Statins + niacin were unrelated to neuropathy. On the whole, the findings of the present study demonstrate that the controversial association of statins with neuropathy may be due to the fact that previous studies have not included the use of niacin and the potential neuroprotective effects of niacin. Multiple reports have stated that niacin is no longer beneficial for the management of hyperlipidemia and should be abandoned. However, given the apparent ability of niacin to reduce the risk of neuropathy, perhaps niacin should not be discarded before further studies are performed to provide more in depth information.

## Introduction

Statins are a class of lipid-lowering medications that reduce illness and mortality in those who are at a high risk of developing cardiovascular disease. They are the most common cholesterol-lowering drugs. A case control study published in 2002 indicated that statins may increase the risk of peripheral neuropathy. Statin users were shown to be 14-fold more likely to develop peripheral neuropathy than non-users, although the overall risk of developing neuropathy was minimal (1).

Using electronic instruments, Emad *et al* found that the sensory and motor wave features (amplitude, latency and nerve conduction velocity) of the peripheral nerves (median, ulnar, tibial, sural and peroneal) in statin users were aberrant (2). However, a number of other studies have produced conflicting results regarding neuropathy and statins [summarized in (3)].

Statins are frequently combined with niacin (vitamin B3). Due to its beneficial effects on lipid profiles, niacin has been prescribed for the prevention of heart disease for >40 years (4). B vitamins play important roles as coenzymes in the nervous system. A number of neurological diseases have been shown to be associated with deficiencies in one or more B vitamins, and B vitamins can improve certain neurological conditions even without a definite deficiency (5). Among the B vitamins, niacin has long been recognized as a key mediator of neuronal development and survival (6) and may be of value for the treatment of neuropathy (7).

Therefore, the present study aimed to assess whether the combination of niacin and statin may be used to reduce the risk of peripheral neuropathy attributed to statins.

## Data and methods

### Data collection

The present study analyzed data from MedWatch, the Food and Drug Administration (FDA) Safety Information and Adverse Event Reporting Program (8,9). MedWatch was organized in 1993 to collect data regarding adverse events in healthcare. An adverse event is any undesirable experience associated with the use of a medical product. The MedWatch system collects reports of adverse reactions and quality problems, primarily due to drugs and medical devices, but also for other FDA-regulated products (e.g., dietary supplements, cosmetics, medical foods and infant formulas).

MedWatch offers a selection between a voluntary reporting form, designed primarily for health care professionals and the general public, and a mandatory adverse event reporting service (AERS) form, available to manufacturers, importers, and medical product user facilities that manage and store medical products. The latter group is required by law to submit the mandatory form immediately upon the discovery of a product malfunction. Printable mail-in forms are available as an alternative to the online submission system (10).

A MedWatch report of an adverse event does not establish causation. For any given report, there is no certainty that the drug in question caused the reaction. The adverse event may have been related to the underlying disease being treated, another drug being taken concurrently, or something else.

Machine-readable data from MedWatch, including adverse drug reaction reports from manufacturers, are part of a public database. In the present study, the online tool OpenVigil 2.1 was used to query the database (11,12). OpenVigil data are exclusively from FDA and MedWatch, not from social media (13). OpenVigil calculates proportional reporting ratios (PRRs) from adverse drug reaction reports to determine whether the combination of a drug and adverse event are related, using the criteria presented in the study by Evans *et al* (14). A PRR=2 indicates that the adverse reaction is 2-fold more frequent in users of the drug than in the general population. According to the criteria presented in the study by Evans *et al* (14), n>3 adverse events, Chi-squared values with Yates correction >4 (P=0.05), and PRR >2 indicate that the adverse reaction and the drug are related.

The MedWatch data are imperfect, with under- and over-reporting, missing denominator (that is, the number of doses for a drug), wrong, duplicate and/or missing data in the database (11). Consequently, the total number of adverse event reports for all drugs and/or the drug in question from OpenVigil can vary slightly from drug to drug and for different adverse events related to the same drug. The flawed MedWatch data have presented a problem that all analytical software programs, such as OpenVigil, have been forced to confront (15).

FDA has issued MedWatch disclaimers: Rates of occurrence cannot be established with reports. The number of suspected reactions in the FDA Adverse Event Reporting System (FAERS) should not be used to determine the likelihood of a side-effect occurring. FDA does not receive reports for each adverse event or medication error that occurs with a product. A number of factors can determine whether an event will be reported, such as the time a product has been marketed and publicity regarding an event. Therefore, the information in these reports cannot be used to estimate the incidence (occurrence rates) of the reactions reported. Importantly, the FAERS data alone are not an indicator of the safety profile of the drug (https://www.fda.gov/drugs/surveillance/questions-and-answers-fdas-adverse-event-reporting-system-faers).

### Statistical analysis

The Chi-squared test with Yates correction was used to determine whether an adverse reaction and drug were related. The 2-tailed Fisher’s exact test was used to evaluate the differences in reports of neuropathy between statins alone and statins in combination with niacin.

## Results

The data used for the evaluation of the criteria presented in the study by Evans *et al* (14) for neuropathy (n>3 adverse events, Chi-squared test value >4, PRR >2) are presented in Tables I and II for statins alone, and in Table III for statins + niacin. OpenVigil has calculated PRRs from adverse drug reaction reports to determine whether the combination of drug and adverse event are related. Table II indicates statin association with neuropathy (yes or no), determined by Open Vigil 2.1, as well as statin solubility.

The majority of statins alone were related to neuropathy. The association with neuropathy was most pronounced in the lipophilic statins, atorvastatin and fluvastatin. The association was weaker for the other lipophilic statins, lovastatin and simvastatin, possibly since lovastatin and simvastatin are less potent than atorvastatin and fluvastatin. Two hydrophilic statins, rosuvastatin and pravastatin, had a similarly weaker association to neuropathy than atorvastatin and fluvastatin, while pitavastatin had no reports of neuropathy. ‘All other drugs’ refers to all drugs in the FDA MedWatch database. Of the 291,617 reports of statin side-effects to FDA, 218 were of neuropathy, 0.07% (Table I).

Statins + niacin were unrelated to neuropathy. A total of 3 reports of neuropathy were submitted to FDA for patients on this drug combination, of 16,270 reports of statin + niacin side effects, 0.02% (Table III).

The difference in reports of neuropathy between statins alone and statins + niacin (0.07% vs. 0.02%; Table IV) was significant (P=0.006, 2-sided Fisher’s exact test). MedWatch does not record dose.

## Discussion

Common statin-related side-effects (headaches, stomach upset, abnormal liver function tests and muscle cramps) are more common in lipophilic statins. Hydrophilic statins, pitavastatin in particular, seem to have fewer muscle side-effects than statins that are lipid-soluble (16).

To enter the nervous system and cross the blood brain barrier, drugs must be lipophilic and of low molecular weight (17). Among the statins analyzed in the present study, the association of neuropathy with lipophilicity was notable, particularly in the case of atorvastatin. Pitavastatin, pravastatin and rosuvastatin, all hydrophilic, exhibited no association or a lesser association with neuropathy.

Peripheral neuropathy is a credible side-effect of statins. Due to their inhibition of cholesterol synthesis, statins may alter the function and integrity of nerve cell membranes, in which cholesterol plays an important role. Statin-induced neuropathy may be related to the inhibition of HMG Co-A reductase, the enzyme producing mevalonate and ultimately cholesterol. In addition, statins inhibit the mitochondrial enzyme ubiquinone (coenzyme Q10) which plays a crucial role in the mitochondrial respiratory process that controls energy use in nerve and striated muscle tissue (2). The administration of coenzyme Q10 may prevent or diminish neuromuscular damage from statins, as has been shown for other complications. Pharmaceutical manufacturers have long been aware of this possibility, and Merck & Co., has tested a statin-coenzyme Q10 combination (18).

Peripheral neuropathy is a rare complication associated with the use of statins (19). Gaist *et al* (1), reported that for those aged ≥50, one excess case of idiopathic peripheral neuropathy occurred for every 2,200 (95% CI, 880 to 7,300) person-years of statin use. The neuropathy had electrophysiologic features predominantly of axonal degeneration, and usually presented with pain, paraesthesias and numbness; muscle stretch reflexes were absent in half of the cases. The analysis did not measure the severity of the peripheral neuropathy.

Koslik *et al* (20), investigated neuropathy and related adverse effects (muscle, cognitive functions and fatigue) in a group of physicians on statins. An older age was a risk factor and a higher dose was associated with an increased risk. Not all cases had resolution of symptoms following the discontinuation of statins.

The association of statins with neuropathy continues to generate controversy. For example, the large Fremantle Diabetes Study suggested that statins may exert neuroprotective effects in neurological disorders, such as Alzheimer disease, Parkinson’s disease, multiple sclerosis and primary brain tumors (21,22). In addition to their potent anti-atherosclerotic and cardioprotective effects, compelling clinical and preclinical studies delineate the neuroprotective efficacy of statins in all these neurological disorders.

The administration of niacin is associated with a number of side-effects, itching, flushing and fatigue, particularly at higher doses, thus making its use difficult. Yet only a small dose of niacin may be required to reduce risk of neuropathy in statin users.

The controversial association between statins and neuropathy may be because previous studies have not analyzed the use of niacin and the potential neuroprotective effects of niacin. An alternative explanation may be that individuals who can tolerate the side-effects of niacin can also tolerate neuropathy and thus do not report it.

Multiple reports have stated that niacin is no longer useful for the management of hyperlipidemia and should be abandoned (23). However, given the apparent ability of niacin to reduce the risk of neuropathy, niacin should perhaps not be discarded just yet. Further studies are required to fully determine the mechanisms of niacin with regard to neuropathy and statin use.

## Figures and Tables

**Table I.** Data used for the evaluation of the criteria presented in the study by Evans *et al* (14) for neuropathy for statins alone.

Drug	Drug Neuropathy	Other events	Total events	%	All other drugs		Total events	Chi-Squared test +Yates	PRR	PRR 95% CI Lower bound	PRR 95% CI Upper bound
					Neuropathy	Other events					

Atorvastatin	113	108,130	108,243	0.104	1,502	7,472,720	7,474,222	352	5.20	4.3	6.3
Fluvastatin	4	3,234	3,238	0.12	1,611	7,57,7616	7,579,227	11.5	5.80	2.2	15.5
Lovastatin	7	9,562	9,569	0.07	1,608	7,571,288	7,572,896	9.8	3.40	1.6	7.2
Simvastatin	54	101,770	101,824	0.05	1,561	7,479,080	7,480,641	47.3	2.50	1.9	3.3
Pitavastatin	0	762	762	0	1,615	7,580,088	7,581,703	0.7	0.00	0	0
Pravastatin	17	22,037	22,054	0.08	1,598	7,558,813	7,560,411	29.7	3.60	2.3	5.9
Rosuvastatin	23	45,904	45,927	0.05	1,592	7,534,946	7,536,538	16.6	2.40	1.6	3.6

According to these criteria (n>3 adverse events, Chi-squared test value >4, PRR >2), the majority of statins alone were associated with neuropathy. Pitavastatin was the single exception, with no cases of neuropathy reported to FDA (column 2). The assocation with neuropathy was most pronounced in the lipophilic statins: Atorvastatin and fluvastatin. The association was weaker for the other lipophilic statins, lovastatin and simvastatin. Two hydrophilic statins, rosuvastatin and pravastatin, had a similarly weaker association to neuropathy. All other drugs refers to all drugs in the FDA MedWatch database. OpenVigil calculates proportional reporting ratios (PRRs) from adverse drug reaction reports to determine whether the combination of drug and adverse event are related.

**Table II.** Statin association with neuropathy.

Drug	Neuropathy	Solubility

Atorvastatin	Yes	Lipophilic
Fluvastatin	Yes	Lipophilic
Lovastatin	Yes	Lipophilic
Simvastatin	Yes	Lipophilic
Pitavastatin	No	Hydrophilic
Pravastatin	Yes	Hydrophilic
Rosuvastatin	Yes	Hydrophilic

Statin association with neuropathy is indicated (yes or no), calculated using Open Vigil 2.1 in Table I. Statin solubility is also indicated.

**Table III.** Data used for the evaluation of the criteria presented in the study by Evans *et al* (14) for neuropathy in patients taking statin + niacin.

Drug	Drug Neuropathy	Other events	Total events	%	All other drugs		Total events	Chi-Squared test +Yates	PRR	PRR 95% CI Lower bound	PRR 95% CI Upper bound
					Neuropathy	Other events					

Atorvastatin	2	2,522	2,524	0.08	1,613	7,578,328	7,579,941	1.7	3.70	0.9	15
Fluvastatin	0	51	51	0	1,615	7,580,799	7,582,414	22	0.00	0	0
Lovastatin	1	987	988	0.1	1,614	7,579,863	7,581,477	0.4	4.80	0.7	34
Simvastatin	0	9,847	9,847	0	1,615	7,571,003	7,572,618	1.2	0.00	0	0
Pitavastatin	0	21	21	0	1,615	7,580,829	7,582,444	55	0.00	0	0
Pravastatin	0	701	701	0	1,615	7,580,149	7,581,764	0.82	0	0	0
Rosuvastatin	0	2,138	2,138	0	1,615	7,578,712	7,580,327	0.004	0	0	0

Statin + niacin were unrelated to neuropathy.

**Table IV.** Differences in reports of neuropathy between the use of statins alone and that of statins + niacin.

	Statins alone	Statins + niacin

Neuropathy	218	3
No neuropathy	291,399	16,267
Total	291,617	16,270

Significant differences were found (0.07 vs. 0.02%; P=0.006, 2-sided Fisher’s exact test, data from Tables I and III).

## References

[1] Gaist D, Jeppesen U, Andersen M, Garcia Rodriguez LA, Hallas J and Sindrup SH: Statins and risk of polyneuropathy: A case-control study. Neurology 58: 1333–1337, 2002. 12011277 10.1212/wnl.58.9.1333

[2] Emad M, Arjmand H, Farpour HR and Kardeh B: Lipid-lowering drugs (statins) and peripheral neuropathy. Electron Physician 10: 6527–6533, 2018. 29765578 10.19082/6527 PMC5942574

[3] Warendorf JK, Vrancken A, van Eijk RPA, Visser NA, van den Berg LH and Notermans NC: Statins do not increase risk of polyneuropathy: A case-control study and literature review. Neurology 92: e2136–e2144, 2019. 30737334 10.1212/WNL.0000000000007148

[4] Hr Superko, Zhao XQ, Hodis HN and Guyton JR: Niacin and heart disease prevention: Engraving its tombstone is a mistake. J Clin Lipidol 11: 1309–1317, 2017. 28927896 10.1016/j.jacl.2017.08.005

[5] Calderon-Ospina CA and Nava-Mesa MO: B Vitamins in the nervous system: Current knowledge of the biochemical modes of action and synergies of thiamine, pyridoxine, and cobalamin. CNS Neurosci Ther 26: 5–13, 2020. 31490017 10.1111/cns.13207 PMC6930825

[6] Gasperi V, Sibilano M, Savini I and Catani MV: Niacin in the central nervous system: An update of biological aspects and clinical applications. Int J Mol Sci 20: 974, 2019. 10.3390/ijms20040974 PMC6412771 30813414

[7] Bolino A, Piguet F, Alberizzi V, Pellegatta M, Rivellini C, Guerrero-Valero M, Noseda R, Brombin C, Nonis A, D’Adamo P, : Niacin-mediated Tace activation ameliorates CMT neuropathies with focal hypermyelination. EMBO Mol Med 8: 1438–1454, 2016. 27799291 10.15252/emmm.201606349 PMC5167133

[8] Kessler DA: Introducing MEDWatch. A new approach to reporting medication and device adverse effects and product problems. JAMA 269: 2765–2768, 1993. 8492403 10.1001/jama.269.21.2765

[9] Lehrer S and Rheinstein PH: Nonsteroidal anti-inflammatory drugs (NSAIDs) reduce suicidal ideation and depression. Discov Med 28: 205–212, 2019. 31928628

[10] Craigle V: MedWatch: The FDA safety information and adverse event reporting program. J Med Library Association 95: 224, 2007.

[11] Bohm R, Hocker J, Cascorbi I and Herdegen T: OpenVigil-free eyeballs on AERS pharmacovigilance data. Nat Biotechnol 30: 137–138, 2012. 22318027 10.1038/nbt.2113

[12] Böhm R, von Hehn L, Herdegen T, Klein HJ, Bruhn O, Petri H and Höcker J: OpenVigil FDA-Inspection of U. S. American Adverse Drug Events Pharmacovigilance Data and Novel Clinical Applications. PLoS. One 11: e0157753, 2016. 27326858 10.1371/journal.pone.0157753 PMC4915658

[13] Coloma PM, Becker B, Sturkenboom MC, van Mulligen EM and Kors JA: Evaluating social media networks in medicines safety surveillance: Two case studies. Drug Saf 38: 921–930, 2015. 26242616 10.1007/s40264-015-0333-5 PMC4579253

[14] Evans SJ, Waller PC and Davis S: Use of proportional reporting ratios (PRRs) for signal generation from spontaneous adverse drug reaction reports. Pharmacoepidemiol Drug Saf 10: 483–486, 2001. 11828828 10.1002/pds.677

[15] Hauben M, Reich L, DeMicco J and Kim K: ‘Extreme duplication’ in the US FDA Adverse Events Reporting System database. Drug Saf 30: 551–554, 2007. 17536881 10.2165/00002018-200730060-00009

[16] Mukhtar RY, Reid J and Reckless JP: Pitavastatin. Int J Clin Pract 59: 239–252, 2005. 15854203 10.1111/j.1742-1241.2005.00461.x

[17] Alavijeh MS, Chishty M, Qaiser MZ and Palmer AM: Drug metabolism and pharmacokinetics, the blood-brain barrier, and central nervous system drug discovery. NeuroRx 2: 554–571, 2005. 16489365 10.1602/neurorx.2.4.554 PMC1201315

[18] Rosch PJ: Peripheral neuropathy. Lancet 364: 1663–1665, 2004. 10.1016/S0140-6736(04)17345-5 15530619

[19] Donaghy M: Assessing the risk of drug-induced neurologic disorders: Statins and neuropathy. Neurology 58: 1321–1322, 2002. 12011272 10.1212/wnl.58.9.1321

[20] Koslik HJ, Meskimen AH and Golomb BA: Physicians’ Experiences as patients with statin side effects: A case series. Drug Saf Case Rep 4: 3, 2017. 28217821 10.1007/s40800-017-0045-0 PMC5316517

[21] Weimer LH and Sachdev N: Update on medication-induced peripheral neuropathy. Curr Neurol Neurosci Rep 9: 69–75, 2009. 19080756 10.1007/s11910-009-0011-z

[22] Malfitano AM, Marasco G, Proto MC, Laezza C, Gazzerro P and Bifulco M: Statins in neurological disorders: An overview and update. Pharmacol Res 88: 74–83, 2014. 24954580 10.1016/j.phrs.2014.06.007

[23] Lloyd-Jones DM: Niacin and HDL cholesterol-time to face facts. N Engl J Med 371: 271–273, 2014. 25014692 10.1056/NEJMe1406410

